# Cuproptosis-Related Gene FDX1 Suppresses the Growth and Progression of Colorectal Cancer by Retarding EMT Progress

**DOI:** 10.1007/s10528-024-10784-8

**Published:** 2024-03-23

**Authors:** Chao Wang, Jingjing Guo, Yun Zhang, Shusheng Zhou, Bing Jiang

**Affiliations:** 1 Department of Internal Medicine Oncology, Chaohu Hospital of Anhui Medical University, No. 64, Chaohu North Road, Juchao District, Chaohu, 238000 Anhui China; 2 Department of Gastrointestinal Surgery, Chaohu Hospital of Anhui Medical University, Chaohu, 238000 Anhui China

**Keywords:** FDX1, Colorectal cancer, EMT progress, EGF

## Abstract

Colorectal cancer (CRC) is a usual cancer and a kind of lethiferous cancer. Cuproptosis-related gene ferredoxin 1 (FDX1) has been discovered to act as a suppressor, thereby suppressing some cancers’ progression. But, the regulatory functions of FDX1 in CRC progression keep vague. In this work, at first, through TCGA database, it was revealed that FDX1 exhibited lower expression in COAD (colon adenocarcinoma) tissues, and CRC patients with lower FDX1 expression had worse prognosis. Furthermore, FDX1 expression was verified to be down-regulated in CRC tissues (*n* = 30) and cells. It was further uncovered that FDX1 expression was positively correlated with CDH1 and TJP1 (epithelial marker), and negatively correlated with CDH2, TWIST1, and FN1 (stromal marker), suggesting that FDX1 was closely associated with the epithelial–mesenchymal transition (EMT) progress. Next, it was demonstrated that overexpression of FDX1 suppressed cell viability, invasion, and migration in CRC. Furthermore, it was verified that FDX1 retarded the EMT progress in CRC. Lastly, through rescue assays, the inhibited CRC progression mediated by FDX1 overexpression was rescued by EGF (EMT inducer) treatment. At last, it was uncovered that the tumor growth and metastasis were relieved after FDX1 overexpression, but these changes were reversed after EGF treatment. In conclusion, FDX1 inhibited the growth and progression of CRC by inhibiting EMT progress. This discovery hinted that FDX1 may act as an effective candidate for CRC treatment.

## Introduction

Currently, colorectal cancer (CRC) is the top three tumors for incidence and the second highest for mortality (Baidoun et al. 2021; Dekker et al. 2019). There is about 1.9 million new cancer cases and 0.9 million cancer deaths in 2020 (Sung et al. 2021). Additionally, the incidence of CRC in 2020 still accounts for 10% of the total cancer cases, and 3.2 million new cases have been expected by 2040 (Morgan et al. 2023; Sung et al. 2021). The prognosis of CRC is largely affected by clinicopathological features and tumor stage (Dienstmann et al. 2019). The effective early screening has relieved the incidence of severe CRC patients, and some treatment (such as targeted therapy, immunotherapy, chemotherapy) have contributed to the decline in morbidity and mortality of CRC (Biller and Schrag 2021). However, more than 50% of CRC patients are still diagnosed with advanced stages, seriously threatening their lives (Benson 2007). Now, the mean age of onset is lower, and the 5-year survival rate for CRC patients with distant metastasis is under 10% (Erickson 2018). For obtaining effective treatment strategies, it is needful to conduct investigations into the carcinogenicity of CRC to seek new and promising biomarkers.

Copper death is a novel discovered mechanism of cell death (Jiang et al. 2022). The main mechanism of copper death is that the direct targeting of copper to the fatty acylation component of the tricarboxylic (TCA) cycle, followed by the loss of iron-sulfur cluster protein, which brings about protein toxic stress and ultimately cell death (Tsvetkov et al. 2022). Ferredoxin 1 (FDX1) is a pivotal modulator of copper ion carrier-stimulated cell death and an upstream factor of protein lipidation (Tsvetkov et al. 2022). Knockdown of FDX1 results in a complete loss of protein lipidation, which in turn protects cells against copper toxicity (Sheftel et al. 2010). FDX1 is present in the mitochondrial matrix and owns effects in the biosynthesis of a small iron–sulfur (Fe–S) cluster that transfers electrons from NADPH to mitochondrial cytochrome P450 through ferredoxin reductase, to generate steroid (Sheftel et al. 2010; Strushkevich et al. 2011). Fe–S clusters are evolutionarily conserved in all organisms and own functions in a variety of biochemical processes, containing gene expression modulation, mitochondrial respiratory chains, redox catalysis, and central metabolism (Py and Barras 2010). So that, FDX1 has also been discovered to own important roles to participate into various cancers’ progression through playing as a suppressor (Chen et al. 2023; Jiang et al. 2023; Zhang et al. 2022). Interestingly, one report has uncovered that the expression of FDX1 in colon adenocarcinoma (COAD) tissues is significantly lower than that in normal tissues, and the overall survival rate of COAD patients with FDX1 high expression group is better than that in patients with FDX1 low expression (Wang et al. 2022). These data implied that FDX1 may own key regulatory functions in the pathogenesis of CRC, but the detailed regulatory mechanism of FDX1 in CRC keep largely unknown.

The purpose of this study was to investigate the roles and regulatory mechanisms of FDX1 in CRC progression. Our findings firstly revealed that FDX1 suppressed the growth and progression of CRC by inhibiting EMT progress, which might highlight the regulatory functions of FDX1 in the treatment of CRC.

## Materials and Methods

### Tissue Samples

Thirty pairs of CRC tissues and adjacent normal tissues (as negative control) were gathered from CRC patients in the Chaohu Hospital of Anhui Medical University. No treatment has made in these participators. The signature of the informed consents of all participators was obtained. This work was approved by the Ethics Committee of the Chaohu Hospital of Anhui Medical University (KYXM-202202-006). The collected tissues were kept in liquid nitrogen right away for further experiments.

### Cell lines and Culture

The CRC cell lines (SW480, HT-29, HCT116, and LoVo) and normal colonic epithelial cell line (NCM460, as negative control) were purchased from American Tissue Culture Collection (ATCC, USA). The incubation of these cells was made with Dulbecco’s modified Eagle’s medium (DMEM; Gibco, New York, NY, USA) containing 10% fetal bovine serum (FBS, Gibco, USA) in the moist atmosphere with 5% CO_2_ at 37 °C.

### Cell Transfection

The pcDNA3.1 plasmids targeting FDX1 (pcDNA3.1/FDX1) and pcDNA3.1 plasmids (as negative control) were purchased from Ribobio (Guangzhou, China). Lipofectamine 2000 (Invitrogen, USA) was employed for the transfection of these vectors into SW480 and HT-29 cells. The Control group was the blank treatment. The Epidermal growth factor (EGF, 50 ng/mL, EMT inducer) was utilized for treating CRC cells to induce EMT progress.

### RT-qPCR

The isolation of total RNA from CRC cells was done through using TRIzol reagent (Invitrogen, USA). The transcription of RNA to cDNA was made with the SuperScript™ II Reverse Transcriptase Kit (Invitrogen, USA). The implementation of qRT-PCR was performed with the SYBR Premix Ex Taq™ (Takara, Shanghai, China). Normalizing to β-actin (the internal reference), the relative mRNA expressions were figured up through the 2^−∆∆Ct^ method.

### Western Blot

The isolation of proteins from SW480 and HT-29 cells was made through RIPA lysis buffer (Beyotime, Shanghai, China). Then, the separation of these isolated protein was made with sodium dodecyl sulfate–polyacrylamide gel electrophoresis (SDS-PAGE, 10%), and proteins were moved to polyvinylidene fluoride (PVDF) membranes (Amersham, USA). After blocking, primary antibodies including FDX1 (ab109312, 1/1000, Abcam, Shanghai, China), E-cadherin (ab40772, 1/10000), ZO-1 (ab216880, 1/1000), N-cadherin (ab76011, 1/5000), TWIST1 (ab175430, 1/1000), Fibronectin 1 (ab2413, 1/1000), and β-actin (ab8226, 1 µg/mL) mixed into the membranes were performed for 12 h at 4 °C. Then, the secondary antibody (ab6721, 1/5000, Abcam) was further put into the membranes for 2 h. Post-rinsing, the ECL chemiluminescent detection system (Thermo Fisher Scientific) was applied to assess the protein blots, and ImageJ was utilized for analysis.

### CCK-8 assay

SW480 and HT-29 cells were seeded on the 96-well plate. The supplementation of CCK-8 solution (10 µL, Beyotime, Beijing, China) was done at 48 h. After 4-h incubation, the microplate reader (Bio-Rad Laboratories, Hercules, CA) was employed to assess cell viability at OD value (450 nm).

### Transwell Assay

Transwell chambers (pore size, 8 μM; Corning, NY, United States) pre-coated with Matrigel (BD Biosciences, Franklin Lakes, NJ, United States) were adopted in this assay. The serum-free medium (200 μL) and CRC cells were put into the upper chamber, and the DMEM medium with 20% FBS (600 μL) was put into the lower chamber. After 48 h, these invaded cells were subjected to fixation (4% paraformaldehyde) and staining (0.1% crystal violet). Ultimately, one microscope (Leica, Wetzlar, Germany) was utilized to count the invaded cells.

### Wound Healing Assay

The 6-well plate was utilized for incubating CRC cells to achieve 90% confluence. One 100-μL sterile tip was employed to generate a scratch. At 0 h and 24 h, the Olympus optical microscope was applied to observe the wound healing and count the migration ability.

### In Vivo* Assay*

The male BALB/c nude mice (4–6 week old) were acquired from the Vital River Company (Beijing, China). All experimental procedures have gained the approval of the Animal Care Committee of Beijing Viewsolid Biotechnology Co. LTD (VS2126A00165). The animal experiments were performed in accordance with the U.S. Public Health Service Policy on the Humane Care and Use of Laboratory Animals.

Mice (*n* = 15) were randomly separated into 3 groups (*n* = 5 for each group). The injection of the stably transfected SW480 cells into the right flanks of mice were made. The tumor volume was confirmed every 7 days. After 28 days, mice were sacrificed through cervical dislocation. Then, the collected tumors were weighed, and tumor tissues were cut for IHC assay.

### IHC Assay

The tumor Sects. (4 μm paraffin embedded) were performed the dewaxing and re-hydration. After sealing, the sections and primary antibody Ki67 (ab15580, 5 µg/mL, Abcam, Shanghai, China), E-cadherin (ab231303, 1 µg/mL, Abcam), or N-cadherin (ab207608, 1/500, Abcam) were blended for 12-h incubation at 4 °C and further mixing for appropriate secondary antibody (ab6721, 1/1000, Abcam, Shanghai, China). Next, the sections were made the dyeing by diaminobenzidine (DAB) and re-dyeing by hematoxylin. Lastly, images were obtained under a microscope (Nikon, Tokyo, Japan).

### Statistical Analysis

Data were displayed as the mean ± standard deviation (SD). The statistical analysis was made with SPSS version 20.0 software (SPSS, Chicago, USA) and Prism 9 (GraphPad Software). At least three biological repetitions existed in each experiment. In animal experiments, 5 mice were for each group. Difference in two groups was conducted through the Student’s *t* test (paired or unpaired), and difference in multiple groups was conducted through one-way ANOVA by a Tukey multiple comparisons post hoc test. *p* < 0.05 was set as significant difference.

## Results

### FDX1 Owned Lower Expression in CRC Tissues and Cells

**Fig. 1:**
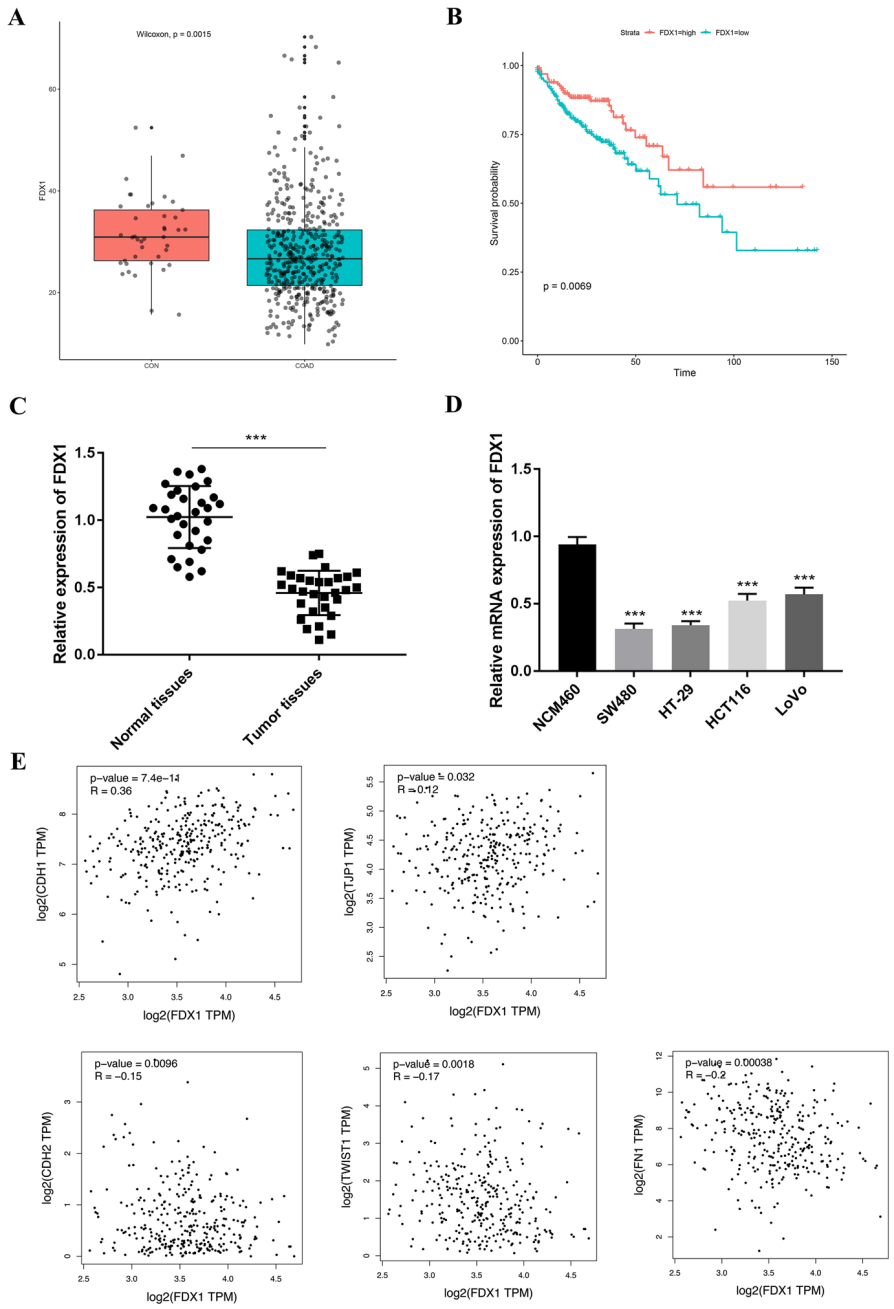
FDX1 expressed lower level in CRC. **A** The expression of FDX1 was verified through TCGA database. **B** The survival probability of CRC patients with high or low FDX1 expression was confirmed through TCGA database. **C** The expression of FDX1 was examined in normal tissues or CRC tissues through RT-qPCR. **D** The FDX1 expression was tested in NCM460, SW480, HT-29, HCT116, and LoVo cells. **E** The correlation between FDX1 and CDH1 (or TJP1, CDH2, TWIST1, FN1) was affirmed. ***p < 0.001

At first, from TCGA database, it was confirmed that the FDX1 expression was down-regulated in COAD (colon adenocarcinoma) tissues (*p* = 0.0015) (Fig. 1A). Additionally, through TCGA database, it was discovered that CRC patients with low FDX1 expression had worse prognosis compared to that with high FDX1 expression (*p* = 0.0069) (Fig. 1B). As shown in Table 1, FDX1 expression was significantly associated with tumor diameter, lymph node metastasis and distant metastasis (*p* < 0.05). Further experiments also demonstrated that the mRNA expression of FDX1 was lower in CRC tissues (*n* = 30) (obtained from CRC patients in the Chaohu Hospital of Anhui Medical University) (*p* < 0.001) (Fig. 1C). Moreover, the mRNA expression of FDX1 was markedly reduced in CRC cells compared with the normal colonic epithelial cell line NCM460 (*p* < 0.001) (Fig. 1D). The SW480 and HT-29 cells were selected for next experiments. It was uncovered that FDX1 expression was positively correlated with epithelial markers CDH1 (*p* = 7.4e−11) and TJP1 (*p* = 0.032) and negatively correlated with stromal markers, CDH2 (*p* = 0.0096), TWIST1 (*p* = 0.0018), and FN1 (*p* = 0.00038) (Fig. 1E). These results revealed that FDX1 owned lower expression in CRC tissues and cells.

**Table 1:** Correlation between FDX1 expression and clinicopathologic characteristics in colorectal cancer patients

Parameters	Number	Low FDX1 (*N* = 15)	High FDX1 (*N* = 15)	*p* value
Age (year)				
≥ 60	11	7	4	0.256
< 60	19	8	11	
Tumor diameter (cm)				
≥ 3	20	14	8	0.013*
< 3	10	1	7	
Gender				
Male	23	11	12	0.666
Female	7	4	3	
Lymph node metastasis				
I+II	14	3	11	0.003*
III+IV	16	12	4	
Distant metastasis				
M0	19	13	6	0.008*
M1	11	2	9	

Categorical variables were compared by the chi-square test

*p < 0.05 was recognized as a significant difference

### Overexpression of FDX1 Suppressed Cell Viability, Invasion, and Migration in CRC

**Fig. 2:**
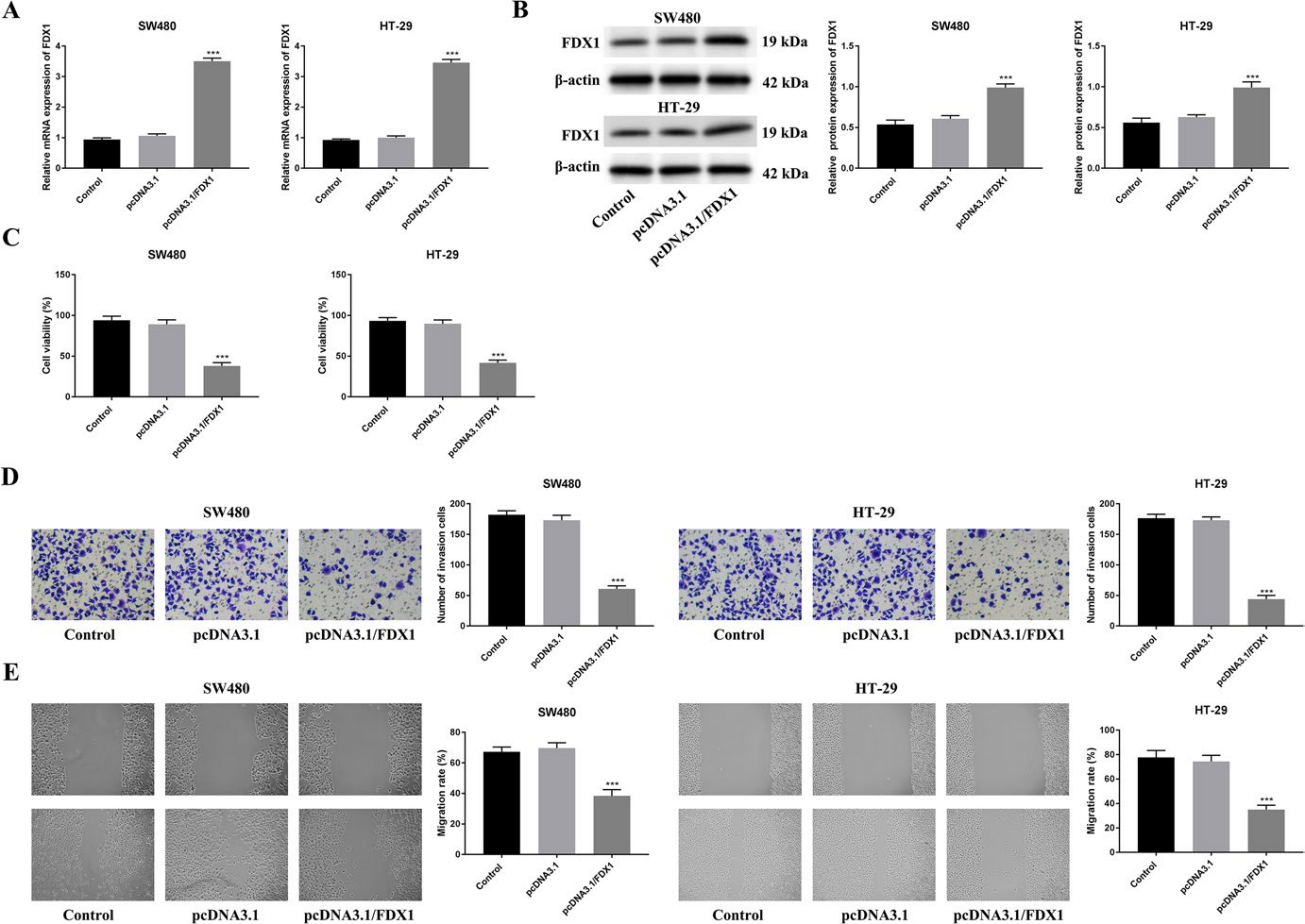
Overexpression of FDX1 suppressed cell viability, invasion, and migration in CRC. **A** The mRNA expression of FDX1 was detected in the Control, pcDNA3.1, and pcDNA3.1/FDX1 groups through RT-qPCR. **B** The protein expression of FDX1 was examined in the Control, pcDNA3.1, and pcDNA3.1/FDX1 groups through western blot. **C** The cell viability was measured in the Control, pcDNA3.1, and pcDNA3.1/FDX1 groups through CCK-8 assay. **D** The cell invasion was tested in the Control, pcDNA3.1, and pcDNA3.1/FDX1 groups through Transwell assay. **E** The cell migration was confirmed in the Control, pcDNA3.1, and pcDNA3.1/FDX1 groups through wound healing assay. ***p < 0.001

The overexpression efficiency of FDX1 was verified in Fig. 2A, B through RT-qPCR and western blot, and FDX1 expression was markedly up-regulated after FDX1 overexpression (*p* < 0.001). Moreover, cell viability was reduced after overexpressing FDX1 (Fig. 2C). The cell invasion was decreased after enhancing FDX1 (*p* < 0.001) (Fig. 2D). In addition, the migration ability was weakened after FDX1 amplification (*p* < 0.001) (Fig. 2E). Taken together, overexpression of FDX1 suppressed cell viability, invasion, and migration in CRC.

### FDX1 Retarded the EMT Progress in CRC

**Fig. 3:**
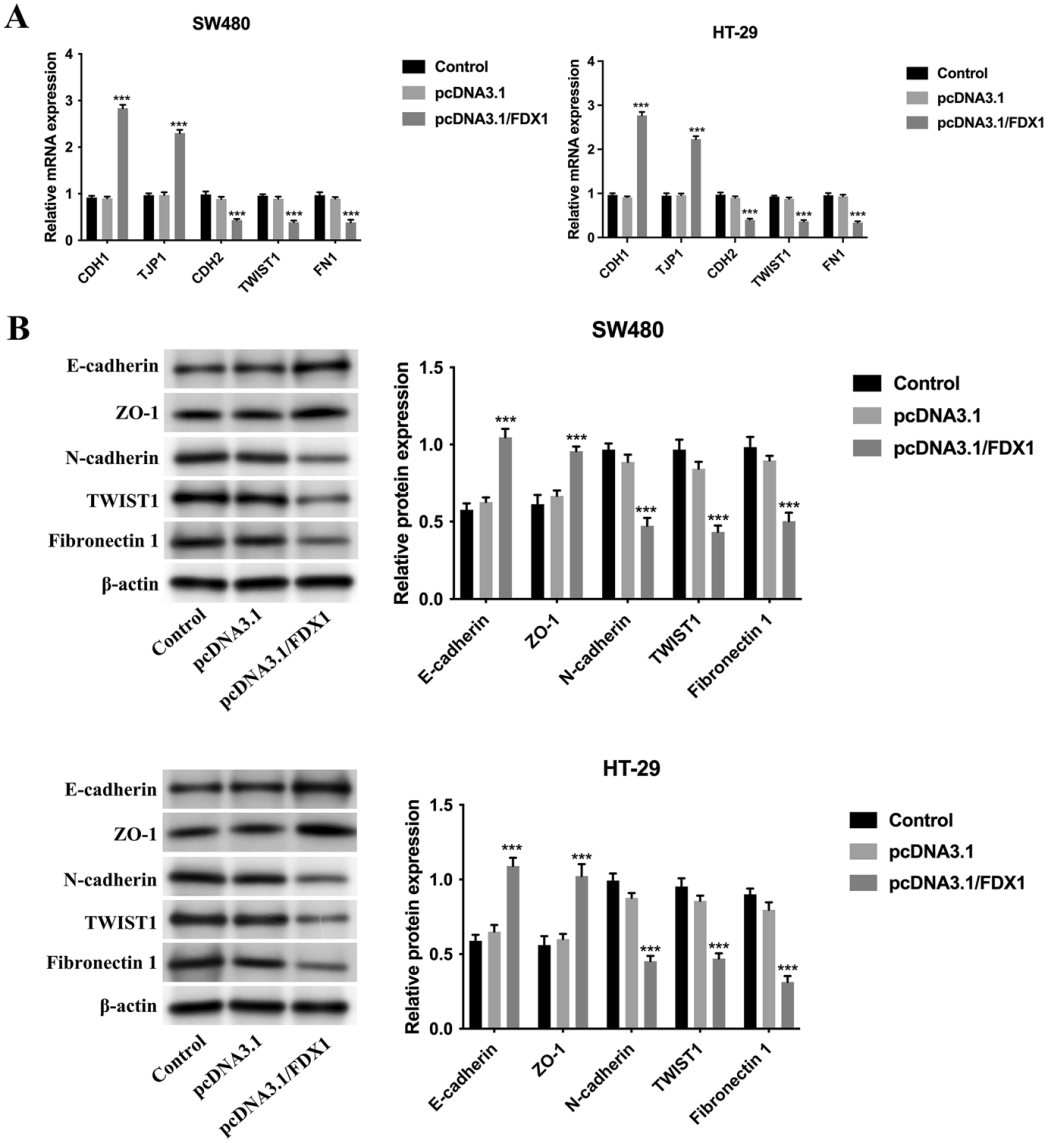
FDX1 retarded the EMT progress in CRC. **A** The mRNA expressions of CDH1, TJP1, CDH2, TWIST1, and FN1 were examined in the Control, pcDNA3.1, and pcDNA3.1/FDX1 groups through RT-qPCR. **B** The protein expressions of E-cadherin, ZO-1, N-cadherin, TWIST1, and Fibronectin 1 were tested in the Control, pcDNA3.1, and pcDNA3.1/FDX1 groups through western blot. ***p < 0.001

The EMT progress is a vital progress in multiple cancers’ progression. So, the regulatory effects of FDX1 on the EMT progress in CRC were further investigated. EMT progress-related genes [CDH1 (encoding protein E-cadherin), TJP1 (encoding protein ZO-1), CDH2 (encoding protein N-cadherin), and TWIST1, FN1 (encoding the protein Fibronectin 1)] were evaluated. The mRNA expressions of CDH1 and TJP1 were increased, and the mRNA expression of CDH2, TWIST1, and FN1 were decreased after up-regulating FDX1 (*p* < 0.001) (Fig. 3A). Similarly, the protein expressions of E-cadherin and ZO-1 were strengthened, and the protein expression of N-cadherin, TWIST1, and Fibronectin 1 was attenuated after overexpressing FDX1 (*p* < 0.001) (Fig. 3B). In a word, FDX1 retarded the EMT progress in CRC.

### The Inhibited CRC Progression Mediated by FDX1 Overexpression was Rescued by EGF (EMT Inducer) Treatment

**Fig. 4:**
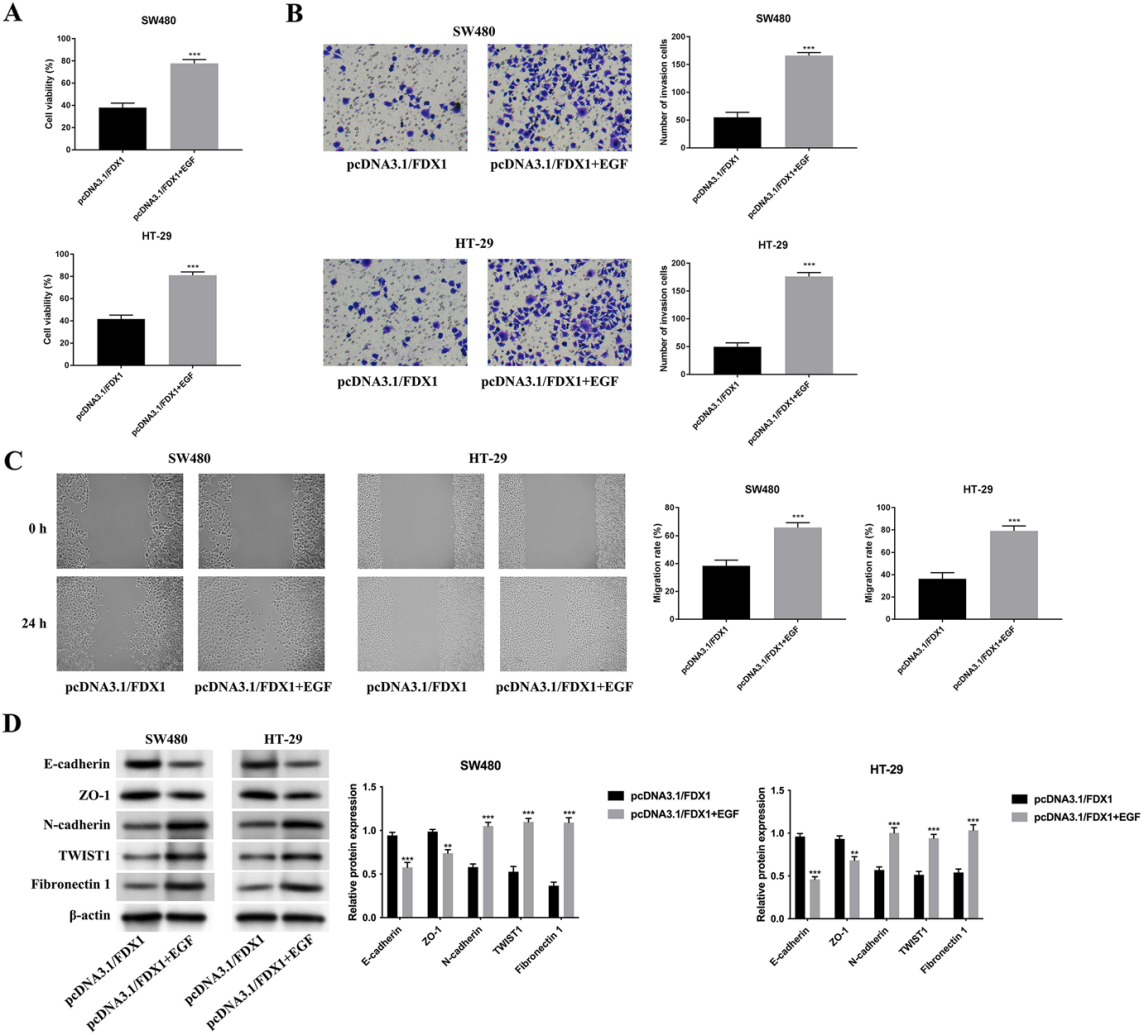
The inhibited CRC progression mediated by FDX1 overexpression was rescued by EGF (EMT inducer) treatment. **A** The cell viability was detected in the pcDNA3.1/FDX1 and pcDNA3.1/FDX1+EGF groups through CCK-8 assay. **B** The cell invasion was tested in the pcDNA3.1/FDX1 and pcDNA3.1/FDX1+EGF groups through Transwell assay. **C** The cell migration was verified in the pcDNA3.1/FDX1 and pcDNA3.1/FDX1+EGF groups through wound healing assay. **D** The protein expressions of E-cadherin, ZO-1, N-cadherin, TWIST1, and Fibronectin 1 were examined in the pcDNA3.1/FDX1 and pcDNA3.1/FDX1+EGF groups through western blot. **p < 0.01 and ***p < 0.001

The rescue assays were further done to confirm the impacts of FDX1 on EMT progress. The reduced cell viability mediated by FDX1 overexpression was counteracted after EGF (50 ng/mL, EMT activator) treatment (*p* < 0.001) (Fig. 4A). In addition, the weakened cell invasion and migration abilities mediated by FDX1 augmentation were rescued after EGF treatment (*p* < 0.001) (Fig. 4B, C). The increased E-cadherin, ZO-1 protein expressions, as well as the decreased N-cadherin, TWIST1, Fibronectin 1 protein expressions induced by FDX1 overexpression were reversed after EGF treatment (*p* < 0.01) (Fig. 4D). These data revealed that the inhibited CRC progression mediated by FDX1 overexpression was rescued by EGF (EMT inducer) treatment.

### *FDX1 Alleviated Tumor Growth and Metastasis *In Vivo* Through Regulating EMT Progress*

**Fig. 5:**
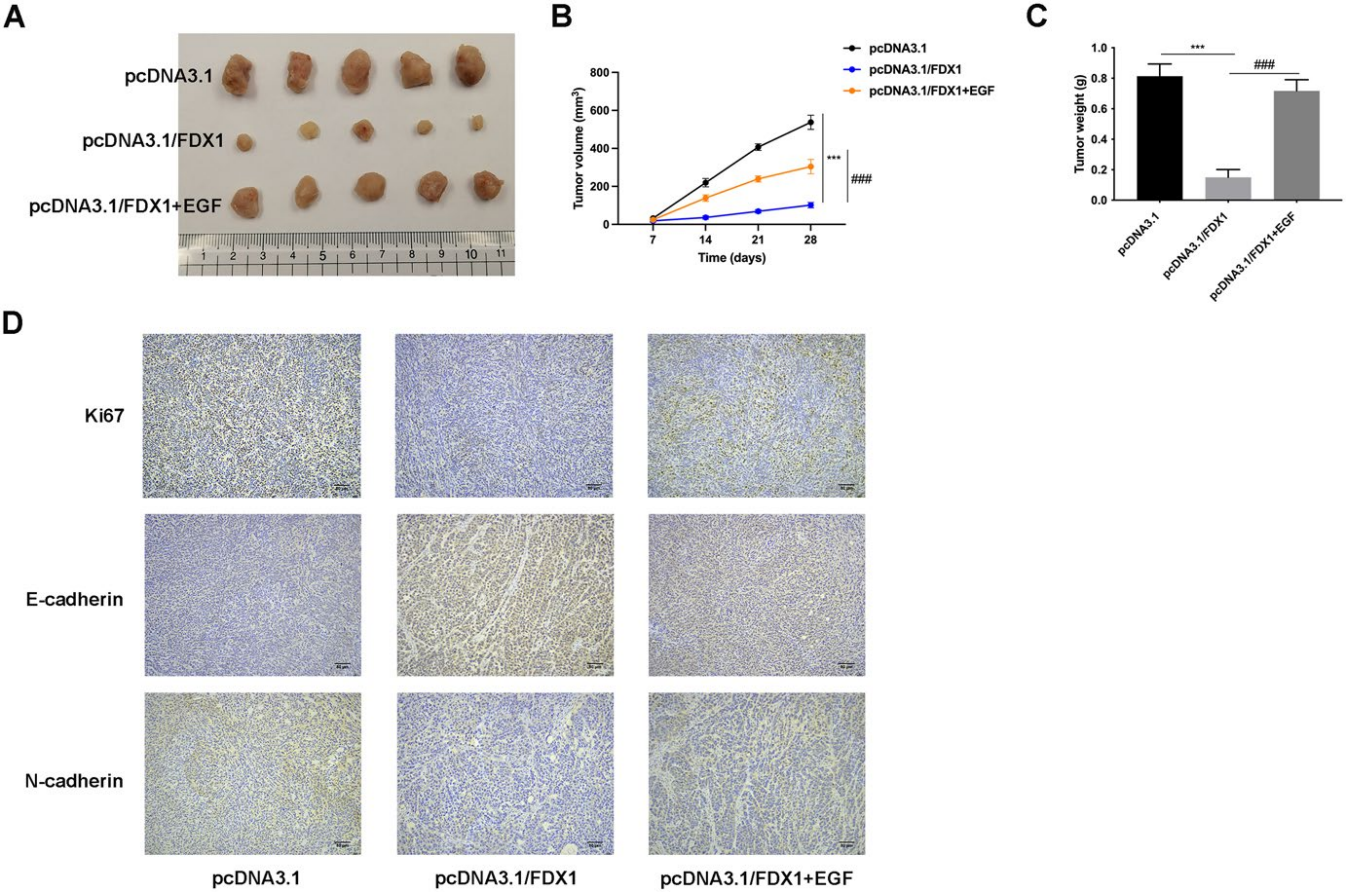
FDX1 relieved tumor growth and metastasis in vivo. **A**–**C** The tumor size, volume, and weight were measured in the pcDNA3.1, pcDNA3.1/FDX1, and pcDNA3.1/FDX1+EGF groups. **D** The protein expressions of Ki67, E-cadherin, and N-cadherin were examined in the pcDNA3.1, pcDNA3.1/FDX1, and pcDNA3.1/FDX1+EGF groups through IHC assay. ***p < 0.001 vs the pcDNA3.1 group; ^###^*p* < 0.001 vs the pcDNA3.1/FDX1 group

Lastly, in vivo experiments were conducted. The tumor size, volume, and weight were all relieved after FDX1 overexpression, but these changes were reversed after EGF treatment (*p* < 0.001) (Fig. 5A–C). Moreover, the Ki67 and N-cadherin expressions were decreased, and E-cadherin expression was increased after FDX1 overexpression, but these effects were rescued after EGF treatment (*p* < 0.001) (Fig. 5D). To sum up, FDX1 relieved tumor growth and metastasis in vivo through regulating EMT progress.

## Discussion

Abundant studies have confirmed that various proteins take part into the development of CRC. For instance, TRIM67 repressed CRC initiation and development through regulating p53 (Wang et al. 2019). Additionally, RNA-binding protein HuR enhances CDC6 to affect oxaliplatin resistance of CRC (Cai et al. 2019). TRIM47 affects the ubiquitination and degradation of SMAD4 to aggravate CRC progression (Liang et al. 2019). Besides, AGR3 regulates Wnt/β-catenin pathway to accelerate the stemness of CRC (Chi et al. 2020). FDX1 has been discovered to own important roles to participate into various cancers’ progression through acting as a suppressor (Chen et al. 2023; Jiang et al. 2023; Zhang et al. 2022). Importantly, FDX1 has been revealed to exhibit lower expression in COAD and affect prognosis (Wang et al. 2022). Similar to this previous report, in this work, at first, from TCGA database, FDX1 exhibited lower expression in COAD tissues, and it was further verified that FDX1 expression was down-regulated in CRC tissues and cells. Moreover, CRC patients with high FDX1 expression had worse prognosis. Next, it was discovered that overexpression of FDX1 suppressed cell viability, invasion, and migration in CRC.

Activating epithelial–mesenchymal transition (EMT) is a pivotal process in tumor metastasis, in which epithelial cells gain mesenchymal cell characteristics and strengthen cell motility and migration capacity (Chen et al. 2022; Pastushenko and Blanpain 2019). The EMT progress has also been affirmed to be a critical progress in CRC progression. For example, PHLDA2 modulates the PI3K/AKT pathway to affect EMT progress and autophagy in CRC (Ma et al. 2020). CAFs secreted exosomes strengthen cell stemness and EMT progress to accelerate metastasis and chemotherapy resistance in CRC (Hu et al. 2019). Moreover, IL-6R/STAT3/miR-34a feedback loop contributes to EMT-mediated invasion and metastasis in CRC (Rokavec et al. 2014). In addition, PRMT5 modulates the EGFR/Akt/GSK3β cascades to affect CRC cell growth and EMT progress (Yan et al. 2021). Besides, KLK8 activated EMT progress in CRC to facilitate cell proliferation and metastasis (Hua et al. 2021). However, the regulatory effects of FDX1 on EMT progress keep unclear and need more investigations.

Current reports have uncovered that copper directly targets the lipid acylated components of the TCA cycle to stimulate copper poisoning, and the accumulation of intermediate metabolites of the TCA cycle in tumor cells can facilitate EMT progress (Cobine and Brady 2022; Georgakopoulos-Soares et al. 2020; Li et al. 2015). Thus, we speculated that cuproptosis-related gene FDX1 may affect TCA cycle to retard EMT progress, thereby suppressing the tumor growth and progression of CRC. In this work, through TCGA database, it was uncovered that FDX1 expression was positively correlated with CDH1 and TJP1 (epithelial marker) and negatively correlated with CDH2, TWIST1, and FN1 (stromal marker), suggesting that FDX1 was closely associated with the EMT progress. Next, experiments also confirmed that FDX1 retarded the EMT progress in CRC. Next, through rescue assays, the inhibited CRC progression mediated by FDX1 overexpression was rescued by EGF (EMT inducer) treatment. At last, our results demonstrated that the tumor growth and metastasis were relieved after FDX1 overexpression, but these changes were rescued after EGF treatment.

In conclusion, for the first time, our work manifested that FDX1 retarded EMT progress to suppress the tumor growth and progression of CRC. The discovery might disclose a novel biomarker-FDX1 and that owns potential important clinical significance in CRC diagnosis and treatment. However, some limitations also exhibit in this study, such as lacking more human samples, animal models, other related regulatory networks (ceRNA, methylation, acetylation, and so on), and other cellular progresses (autophagy, stemness, immune escape, and so on). In future, more experiments were conducted to further seek the other regulatory roles of FDX1 in CRC.

## Data Availability

All the data used to support the findings of this study are included within the article.
